# Role of acid-sensing ion channel 3 in sub-acute-phase inflammation

**DOI:** 10.1186/1744-8069-5-1

**Published:** 2009-01-07

**Authors:** Yi-Tin Yen, Pan-Hsien Tu, Chien-Ju Chen, Yi-Wen Lin, Sung-Tsang Hsieh, Chih-Cheng Chen

**Affiliations:** 1 Institute of Biomedical Sciences, Academia Sinica, Taipei 115, Taiwan; 2 Institute of Zoology, College of Life Science, National Taiwan University, Taipei 106, Taiwan; 3 Department of Anatomy and Cell Biology, National Taiwan University College of Medicine, Taipei 100, Taiwan; 4 Department of Neurology, National Taiwan University Hospital, Taipei 100, Taiwan

## Abstract

**Background:**

Inflammation-mediated hyperalgesia involves tissue acidosis and sensitization of nociceptors. Many studies have reported increased expression of acid-sensing ion channel 3 (ASIC3) in inflammation and enhanced ASIC3 channel activity with pro-inflammatory mediators. However, the role of ASIC3 in inflammation remains inconclusive because of conflicting results generated from studies of *ASIC3 *knockout (*ASIC3*^-/-^) or dominant-negative mutant mice, which have shown normal, decreased or increased hyperalgesia during inflammation.

**Results:**

Here, we tested whether ASIC3 plays an important role in inflammation of subcutaneous tissue of paw and muscle in *ASIC3*^-/- ^mice induced by complete Freund's adjuvant (CFA) or carrageenan by investigating behavioral and pathological responses, as well as the expression profile of ion channels. Compared with the *ASIC3*^+/+ ^controls, *ASIC3*^-/- ^mice showed normal thermal and mechanical hyperalgesia with acute (4-h) intraplantar CFA- or carrageenan-induced inflammation, but the hyperalgesic effects in the sub-acute phase (1–2 days) were milder in all paradigms except for thermal hyperalgesia with CFA-induced inflammation. Interestingly, carrageenan-induced primary hyperalgesia was accompanied by an *ASIC3*-dependent *Nav1.9 *up-regulation and increase of tetrodotoxin (TTX)-resistant sodium currents. CFA-inflamed muscle did not evoke hyperalgesia in *ASIC3*^-/- ^or *ASIC3*^+/+ ^mice, whereas carrageenan-induced inflammation in muscle abolished mechanical hyperalgesia in *ASIC3*^-/- ^mice, as previously described. However, *ASIC3*^-/- ^mice showed attenuated pathological features such as less CFA-induced granulomas and milder carrageenan-evoked vasculitis as compared with *ASIC3*^+/+ ^mice.

**Conclusion:**

We provide a novel finding that ASIC3 participates in the maintenance of sub-acute-phase primary hyperalgesia in subcutaneous inflammation and mediates the process of granuloma formation and vasculitis in intramuscular inflammation.

## Background

Inflammation, the complex reaction of the body to harmful stimuli, is often accompanied by redness, swelling, pain and heat. During inflammation, damaged tissues release pro-inflammatory mediators such as bradykinin, serotonin, histamine, nerve growth factor, prostaglandin, neuropeptides and cytokines to activate immune cells and neurons [1]. These factors serve a protective purpose by stimulating the immune system, which causes vasodilatation to allow the exudation of plasma and leukocytes into the surrounding tissues, whereby the harmful stimuli are removed and the injured tissue undergoes repairing. The extravasation of leukocytes and plasma fluid into the tissue accounts for the swelling of the tissue, whereas the increased blood flow is responsible for the heat and redness.

Inflammation also causes tissue acidosis, whereby high concentrations of protons are the direct cause of pain [2,3]. Acid-sensing ion channel 3 (ASIC3) is the most sensitive nociceptive ion channel responding to tissue acidosis [3,4]. During inflammation, lactic acid, arachidonic acid and nitric oxide sensitize ASIC3 [5-7]. Up-regulation of ASIC3 is seen in inflamed human intestine [8] and dorsal root ganglia (DRG) of rodents with inflamed hind paws [9,10].

Two experimental models of inflammation have been widely used in research of pain. Complete Freund's adjuvant (CFA) is composed of an antigen solution of heat-inactivated bacterium, *Mycobacterium tuberculosis*, emulsified in mineral oil, which can potentiate the cell-mediated immune response and the production of immunoglobulins [11]. A single injection of CFA into the plantar surface of the paw induces intense and persistent inflammation at local injection sites and occasionally at distant locations because of its systemic spread [12]. In contrast, carrageenan is thought to produce non-immune-mediated inflammation [13]. A subcutaneous injection of carrageenan induces inflammatory responses initially mediated by mast cells and neutrophils, and then followed by a phagocytic response, which depends on the mobilization of macrophages. The behavior of *ASIC3*^-/- ^mice has been studied largely with the carrageenan inflammation model but with discrepant results [14-17]. Interestingly, these previous studies imply that ASIC3 might be involved in the development of secondary but not primary hyperalgesia produced by inflammation [16,17].

Sensory neurons innervating muscle and those innervating skin are considered to have different properties, and ASIC3 is more likely expressed in the former than in the latter [18]. To reveal the functional role of ASIC3 in inflammation, we systematically studied thermal and mechanical hyperalgesia induced by CFA and carrageenan in the paw and muscle of *ASIC3*^+/+ ^and *ASIC3*^-/- ^mice. Tissue underwent pathological examination and real-time PCR to reveal the gene regulation of ion channels dependent on ASIC3 following inflammation.

## Results

### Behavior tests

*ASIC3*^+/+ ^and *ASIC3*^-/- ^mice did not differ in baseline responses to thermal and mechanical stimuli or responses in left and right paws. No hyperalgesic effect was observed on the contralateral sides of the injections at most times during this study (Additional file 1).

**Figure 1:**
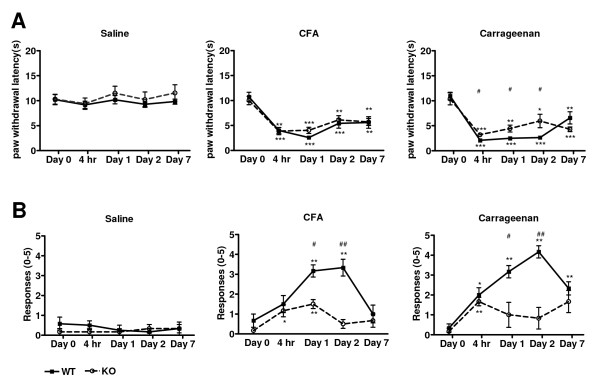
**Intraplantar inflammation-mediated hyperalgesia**. Results of ipsilateral side only. **A**, Thermal hyperalgesia test. **B**, von Frey filament test. WT: *ASIC3*^+/+ ^mice, n = 6 in each group. KO: *ASIC3*^-/- ^mice, n = 6 or 7 in each group. *p < 0.05, **p < 0.01, ***p < 0.001 compared to baseline. #p < 0.05, ##P < 0.01 comparison between *ASIC3*^+/+ ^and *ASIC3*^-/- ^groups. CFA = complete Freund's adjuvant

Thermal hyperalgesia developed in both *ASIC3*^+/+ ^and *ASIC3*^-/- ^mice as soon as 4 h after injection of CFA or carrageenan into paws of both genotypes (Fig. 1A), which persisted for 7 days for both inflammatory models. Although both *ASIC3*^+/+ ^and *ASIC3*^-/- ^mice showed increased sensitivity to heat, the latter showed longer withdrawal latency from 4 h to 2 days than the former in the carrageenan inflammatory model. The difference was small but significant after 4 h, with a mean latency of 3.20 sec and 2.09 sec for *ASIC3*^-/- ^and *ASIC3*^+/+ ^mice, respectively (P < 0.05). On days 1 and 2, paw withdrawal latency for *ASIC3*^+/+ ^mice remained short (mean latency 2.49 sec for day 1 and 2.66 sec for day 2), but the magnitude of hyperalgesia was significantly lower for *ASIC3*^-/- ^mice (mean latency 4.44 sec for day 1 and 5.96 sec for day 2 than that of *ASIC3*^+/+ ^mice (P < 0.05).

Neither *ASIC3*^+/+ ^nor *ASIC3*^-/- ^mice showed a response to von Frey filaments following saline injection, but both developed mechanical hyperalgesia in CFA and carrageenan inflammatory models; however, the mechanical hyperalgesia was much milder and more transient in *ASIC3*^-/- ^mice than in *ASIC3*^+/+ ^mice (Fig 1B). Of 5 applications of the filaments, *ASIC3*^+/+ ^mice with CFA-inflamed paws responded 3–4 times on days 1 and 2, whereas *ASIC3*^-/- ^mice responded only 1–2 times and only on day 1. Both *ASIC3*^+/+ ^and *ASIC3*^-/- ^mice with carrageenan-induced inflammation showed hyperalgesia at a comparable level at 4 hr after injection. Hyperalgesia in *ASIC3*^+/+ ^mice continued to increase on days 1 and 2 but decreased in *ASIC3*^-/- ^mice. On day 7, *ASIC3*^+/+ ^mice still showed mechanical hyperalgesia as compared with at baseline but not *ASIC3*^-/- ^mice.

**Figure 2:**
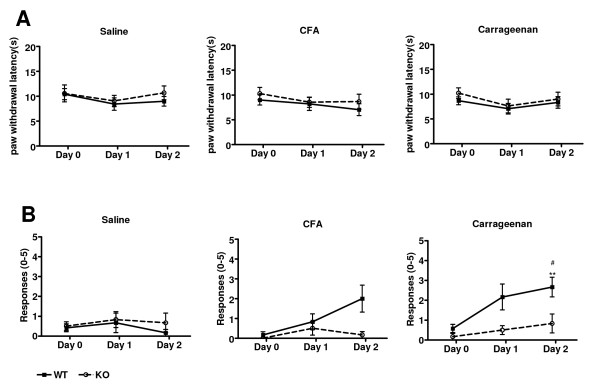
**Intramuscular inflammation-mediated hyperalgesia**. Results of ipsilateral side only. **A**, Thermal hyperalgesia test. **B**, von Frey filament test. WT: *ASIC3*^+/+ ^mice, n = 6 in each group. KO: *ASIC3*^-/- ^mice, n = 6 in each group. **p < 0.01 compared to baseline. #p < 0.05 comparison between *ASIC3*^+/+ ^and *ASIC3*^-/- ^groups. CFA = complete Freund's adjuvant

To test whether the *ASIC3 *gene plays a role in intramuscular inflammation, CFA or carrageenan was injected into gastrocnemius muscle. Then thermal and mechanical tests were applied to the ipsilateral paw, not the inflammatory muscular site, to measure the effect of secondary hyperalgesia. Muscular inflammation had no effect on paw sensitivity to heat stimuli in both genotypes of mice, regardless of the inflammatory agents (Fig. 2A). In *ASIC3*^+/+ ^mice, no significant increase in mechanical sensitivity was observed with CFA-induced inflammation throughout the test period or carrageenan-induced inflammation on day 1. However, mechanical hyperalgesia developed on day 2 in carrageenan-injected *ASIC3*^+/+ ^mice (Fig. 2B) but did not develop in *ASIC3*^-/- ^mice.

**Figure 3:**
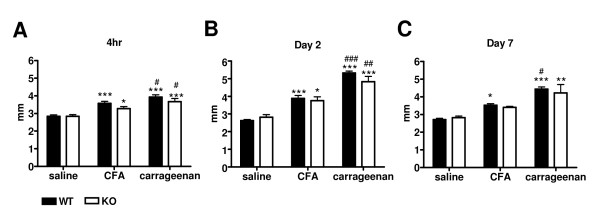
**Paw edema following the induction of inflammation**. Measurement of thickness of ipsilateral paws swelled with inflammation by both complete Freund's adjuvant (CFA) and carrageenan. **A**, Thickness 4 h after intraplantar injection. **B**, Thickness 2 days after injection. **C**, Thickness 7 days after injection. WT: *ASIC3*^+/+ ^mice, n = 6 in each group. KO: *ASIC3*^-/- ^mice, n = 6 in each group. *p < 0.05, **p < 0.01, ***p < 0.001 compared with the saline control group. #p < 0.05, ##p < 0.01, ###p < 0.001 comparison between CFA and carrageenan in the same genotype group.

**Figure 4:**
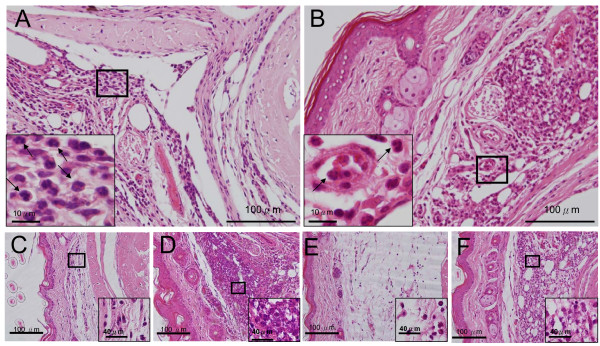
**H&E sections of CFA-inflamed hind paws**. Representative H&E sections obtained 4 hr, 2 and 7 days after intraplantar injection of CFA. **A**, CFA-inflamed *ASIC3*^+/+ ^hind paw on day 2. **B**, CFA-inflamed *ASIC3*^-/- ^hind paw on day 2.**C**, CFA-inflamed *ASIC3*^+/+ ^hind paw at 4 hr. **D**, CFA-inflamed *ASIC3*^+/+ ^hind paw on day 7. **E**, CFA-inflamed *ASIC3*^-/- ^hind paw at 4 hr. **F**, CFA-inflamed *ASIC3*^-/- ^hind paw on day 7. Neutrophils are indicated by black arrows.

**Figure 5:**
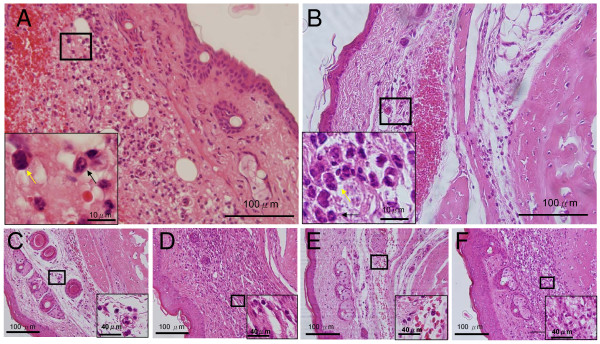
**H&E sections of carrageenan-inflamed hind paws**. Representative H&E sections obtained 4 hr, 2 and 7 days after intraplantar injection of carrageenan. **A**, Carrageenan-inflamed *ASIC3*^+/+ ^hind paw on day 2. **B**, Carrageenan-inflamed *ASIC3*^-/- ^hind paw on day 2. **C**, Carrageenan-inflamed *ASIC3*^+/+ ^hind paw at 4 hr. **D**, Carrageenan -inflamed *ASIC3*^+/+ ^hind paw on day 7. **E**, Carrageenan-inflamed *ASIC3*^-/- ^hind paw at 4 hr. **F**, Carrageenan-inflamed *ASIC3*^-/- ^hind paw on day 7. Neutrophils are indicated by black arrows and mononuclear cells are indicated by yellow arrows.

Examination of pathological specimensTo associate the previously mentioned behavioral changes induced by pain as a result of inflammatory states in these mice, we performed histopathological examination of tissue changes in these samples. For intraplantar injections, remarkable paw edema and redness occurred as soon as 4 h after injection and persisted for 7 days, with variations in the appearance of these inflammatory sites. In 2 of 6 *ASIC3*^+/+ ^mice injected with carrageenan, the inflammation was so severe that black bruises, indicative of subcutaneous hemorrhage in the paw, became evident. Measurement of paw thickness 4 h and 2 and 7 days after the injections of CFA or carrageenan showed significant swelling, with carrageenan causing swelling at all time points (Fig. 3); however *ASIC3*^+/+ ^and *ASIC3*^-/- ^mice did not differ in paw thickness. Histological examination revealed edema and mixed inflammatory infiltration composed of neutrophils and mononuclear inflammatory cells in paw sections of both mice (Fig. 4 &5). The inflammatory infiltration was evident at all time points but was milder at 4 h than at days 2 and 7 (Fig. 4C–F & Fig. 5C–F).

**Figure 6:**
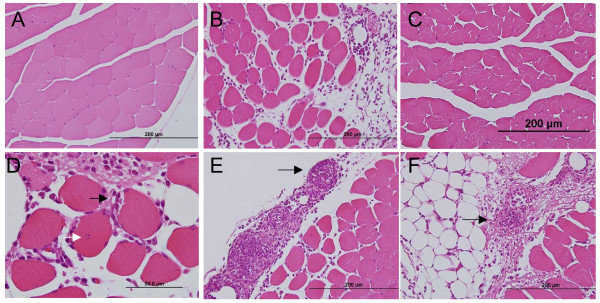
**CFA-inflamed muscle**. Representative H&E sections obtained 2 days after induced inflammation. **A**, Normal control muscle section. **B**, CFA-inflamed *ASIC3*^+/+ ^muscle. Myocytes were separated and rounded up with leukocytes migrated into the intercellular space. Lipid drops surrounded by mononuclear cells were seen. **C**, CFA-inflamed *ASIC3*^-/- ^muscle. **D**, A higher magnitude picture taken from CFA-inflamed *ASIC3*^+/+ ^muscle. Black arrow shows a myocyte being invaded by mononuclear cells. White arrow indicates the centralized nucleus in a myocyte. **E**, Well-formed granulomas (black arrow) in CFA-inflamed *ASIC3*^+/+ ^muscle. **F**, An ill-defined granuloma (black arrow) in CFA-inflamed *ASIC3*^-/- ^muscle.

**Figure 7:**
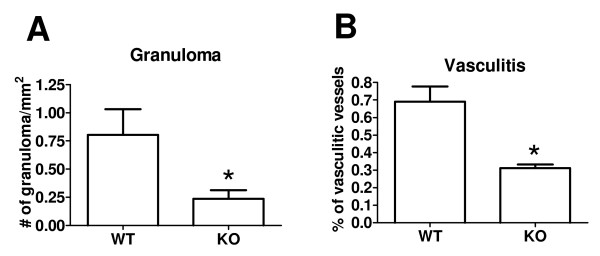
**Comparison of inflammatory features of muscle of *ASIC3*^+/+ ^and *ASIC3*^-/- ^mice at day 2**. **A**, Density of well-formed granuloma was significantly higher in *ASIC3*^+/+ ^(WT) mice than in *ASIC3*^-/- ^(KO) mice (n = 4). **B**, The proportion of vasculitic vessels to total vessels was significantly greater in *ASIC3*^+/+ ^mice (n = 4) than in *ASIC3*^-/- ^mice (n = 5). *p < 0.05

Gross examination of the injection sites of normal saline and CFA was unremarkable, but large areas of hemorrhage could be seen in carrageenan-induced inflamed muscles after skin was removed on day 2. Histologically, the muscle sections of saline groups revealed intact and well-organized muscular fibers, connective tissue and vascular channels with mild infiltration of leukocytes found along the needle tracks in some cases (Fig. 6A). However, marked infiltration of mononuclear inflammatory cells (most likely macrophages) and edema evidenced by the spaces between muscular fibers was observed with CFA injection (Fig. 6B &6C). The inflammatory cells infiltrated endomysial and perimysial connective tissue and caused focal destruction of the myocytes (Fig. 6D). Large round to oval empty spaces representing the deposition of the lipid content from CFA were frequently surrounded by inflammatory infiltrates and the formation of granulomas with epithelioid macrophages was evident (Fig. 6E &6F). CFA contained *M. tuberculosis *and mineral oil, which might well account for the formation of granulomas from day 2. Well-formed granulomas defined by compact, well-circumscribed structures composed of epitheloid macrophages were characteristic of *ASIC3*^+/+ ^mice (Fig 6E), but most of the granulomas found in *ASIC3*^-/- ^mice were small and ill-defined (Fig. 6F). The mean density of granulomas was 0.80 ± 0.23/mm^2 ^for *ASIC3*^+/+ ^mice and 0.24 ± 0.08/mm^2 ^for *ASIC3*^-/- ^mice (p < 0.05) (Fig. 7A). In parallel of this, the myocytes in *ASIC3*^+/+ ^mice showed more severe damage than those in *ASIC3*^-/- ^mice, with more myocytes exhibiting variation in fiber size, centrally located nuclei and invasion by inflammatory cells (Fig. 6B–D). These data indicate that the inflammatory response and subsequent muscular damage induced by CFA injection were milder in the absence of the *ASIC3 *gene.

**Figure 8:**
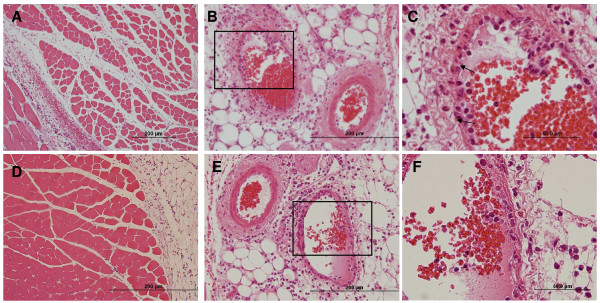
**Carrageenan-inflamed muscle**. Representative H&E sections were obtained 2 days after carrageenan injection. **A-C**, H&E sections from *ASIC3*^+/+ ^mice. **A**, Carrageenan-inflamed ASIC3^+/+ ^muscle. Carrageenan filled in the intercellular matrix, causing inflammation. **B**, Vasculitis in carrageenan-inflamed ASIC3^+/+ ^muscle. The arterial wall (right) was intact, but the vein (left) showed vasculitis. **C**, Higher magnitude of bracket area from **B**. Black granules were deposited on the vascular wall (arrows) and the lining of the lumen was incomplete. **D-F**, H&E sections from *ASIC3*^-/- ^mice. **D**, Carrageenan-inflamed ASIC3^-/- ^muscle. **E**, Vein (right) and artery (left) remained integrated in carrageenan-inflamed ASIC3^-/- ^muscle. **F**, Higher magnitude of bracket area from **E**.

Injection of carrageenan produced a different type of histopathology. Carrageenan appeared as an amorphous pink substance permeating the intercellular space, which induced inflammatory infiltration and extravasation of red blood cells or hemorrhage in severe cases. Inflammatory infiltration into the muscle and subcutaneous fat lobules caused rhabdomyolysis and panniculitis in both *ASIC3*^+/+ ^and *ASIC3*^-/- ^mice (Fig. 8A &8D). Interestingly, inflammatory infiltration was also seen in some of the vascular walls and caused vascular wall destruction or vasculitis (Fig. 8B &8E). The lining of endothelial cells appeared prominent in the damaged areas. Small black granules or nuclear dusts within the disrupted vascular walls were also noted (Fig. 8B &8C). Carrageenan-induced vasculitis might be responsible for the hemorrhage described earlier and contributed to rhabdomyolysis. Interestingly, vasculitis was observed only within veins in this model. Also, the proportion of vessels affected by vasculitis seemed to be higher in *ASIC3*^+/+ ^mice than in *ASIC3*^-/- ^mice (69.02% vs. 31.13% P < 0.05) (Fig. 7B).

**Figure 9:**
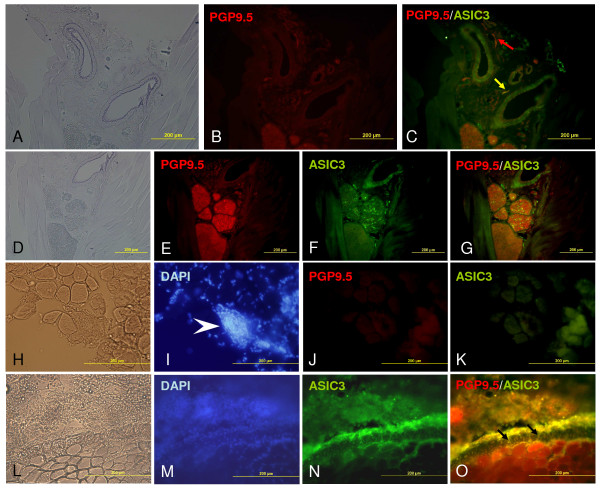
**ASIC3-containing afferents in inflamed muscle**. Red labeling shows secondary antibodies conjugated with Alexa Fluor-594, green with Alexa Fluor-488. **A, B, C**, ASIC3 immunoreactive nerves were found surrounding the vessels and co-localized with PGP9.5. **D, E, F, G**, In nerve bundles, ASIC3 immunoreactivity was co-localized with PGP9.5 (yellow spots in **G**). **H, I, J, K**, ASIC3 and PGP9.5 had no immunoreactivity in areas surrounding granuloma. **L, M, N, O**, ASIC3 and PGP9.5 immunoreactive nerves were found on a vessel with vasculitis. **A, D, H, L**, shows the contrast pictures. Yellow or black arrows indicate the co-localization of ASIC3 and PGP9.5; the red arrow indicates positive staining for PGP9.5 but not ASIC3. The arrowhead shows a well-formed granuloma.

To further identify the precise location of ASIC3-containing afferents in inflamed sites, we stained frozen muscle sections with antibodies against ASIC3 and PGP9.5, which is an ubiqutin C-terminal hydrolase specifically expressed in most neurons [19]. We first confirmed that the ASIC3 immunoreactivity was found in vessels and nerve bundles in non-inflamed areas as previously described (Fig. 9A–G) [18]. In CFA-inflamed muscle, no obvious ASIC3 or PGP9.5 immunoreactivities were found in areas surrounding granuloma (Fig. 9H–K). In contrast, in carrageenan-inflamed muscle, the location of ASIC3 immunoreactivity was often found on vessels with vasuclitis (Fig. 9L–O).

### Gene expression in DRG

To investigate which genes might be involved in the development of hyperalgesia, we examined the transcript levels of *ASIC3*, *Nav1.6*, *Nav1.7*, *Nav1.8*, *Nav1.9 *and *TRPV1 *normalized to *GAPDH *in DRGs of mice 2 days after inflammation induction.

**Figure 10:**
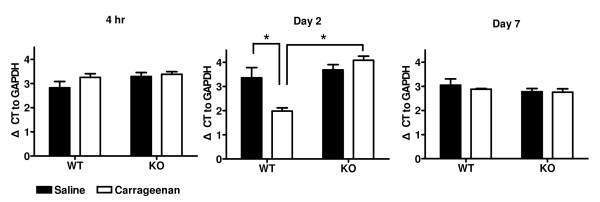
***Nav1.9 *expression in L5 dorsal root ganglia (DRG) 4 hr and 2 and 7 days after intraplantar carrageenan-induced inflammation**. *Nav1.9 *mRNA from the ipsilateral DRG quantified by real-time PCR and normalized to GADPH. (*p < 0.05, n = 3 or 4).

In *ASIC3*^+/+ ^mice, only *Nav1.9 *was up-regulated 2 days after intraplantar carrageenan-induced inflammation (Fig. 10 & Additional file 2). *Nav1.9 *expression with carrageenan injection was significantly increased by twofold as compared with saline injection (mean ΔCT to *GAPDH *3.36 cycles vs. 1.98 cycles, P < 0.05). No up-regulation of *Nav1.9 *could be found in DRGs of mice with CFA-induced inflamed paws and the muscle inflammation models (Additional file 2). This carrageenan-induced up-regulation of *Nav1.9 *was also ASIC3 dependent. In contrast, *Nav1.9 *expression in *ASIC3*^-/- ^mice treated with carrageenan did not differ from that in *ASIC3*^-/- ^mice treated with saline (Fig. 10). The expression of *Nav1.9 *in the carrageenan-treated *ASIC3*^-/- ^group was significantly lower than in the *ASIC3*^+/+ ^group (p < 0.05).

### TTX-resistant sodium currents in DRG neurons

**Figure 11:**
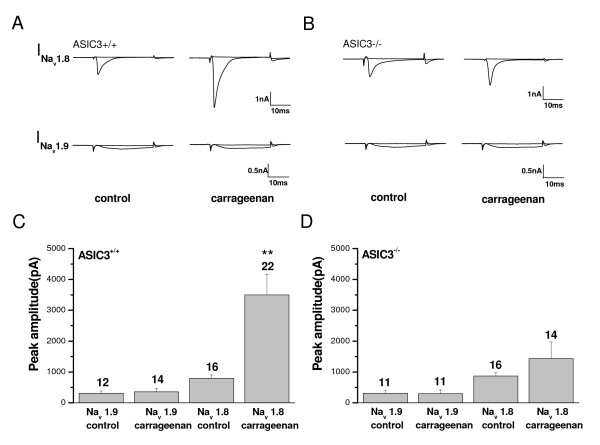
**Tetrodotoxin (TTX)-resistant sodium currents in lumbar DRG neurons**. **A**, Representative TTX-R current traces in *ASIC3*^+/+ ^mice. The TTX-R currents mediated by Nav1.8 (I_Nav1.8_) were induced by membrane depolarization from -80 to -40 mV; TTX-R currents of I_Nav1.9 _were induced membrane depolarization from -80 to -60 mV. **B**, Representative TTX-R current traces in *ASIC3*^-/- ^mice. **C**, Mean peak amplitudes of TTX-R currents in *ASIC3*^+/+ ^mice. **D**, Mean peak amplitudes of TTX-R currents in *ASIC3*^-/- ^mice. The number of experiments (n) is indicated above each bar. **P < 0.01.

To investigate whether null mutation of *ASIC3 *affects the neuronal excitability in DRG neurons during carrageenan-induced inflammation, we conducted whole-cell patch recording to measure the tetrodotoxin (TTX)-resistant (TTX-R) sodium currents (Fig. 11). In small DRG neurons, TTX-R sodium currents are composed of two distinct components: the I_Nav1.8 _activated at about -40 mV and the I_Nav1.9 _activated about -60 mV [20]. In the *ASIC3*^+/+ ^group, I_Nav1.8 _but not I_Nav1.9 _was significantly increased in small-to-medium DRG neurons 2 days after intraplantar carrageenan-induced inflammation (Fig. 11A &11C). However, intraplantar inflammation did not alter the amplitudes of both TTX-R currents in neurons of the *ASIC3*^-/- ^group (Fig. 11B &11D).

## Discussion

### Intraplantar inflammation

Previous studies found that on intraplantar inflammation, *ASIC3*^-/- ^mice exhibited either no or slightly enhanced hyperalgesia [14,15]. Although mouse strains and sexes of these two studies may cause the discrepant results from our current work, the studies used 2% carrageenan, and the tests were carried out only 3–4 h after injection. In another study involving dominant-negative *ASIC3 *transgenic mice [21], behavior tests were performed 1–6 h after zymosan injections to induce paw inflammation. These mice exhibited increased mechanical hypersensitivity. Although the role of ASIC3 in the acute phase of inflammation is inconclusive, our results indicate the involvement of ASIC3 in the sub-acute phase of inflammation. Our study showed no difference in nociceptive behaviors in the acute phase of inflammation (4 h) between two genotypes, except for a small but significant difference in thermal hyperalgesia in the carrageenan inflammatory model. On follow-up in *ASIC3*^-/- ^mice, in both models for mechanical hyperalgesia and in the carrageenan model for thermal hyperalgesia, hyperalgesia was attenuated after intraplantar injection. The inflammation state might have changed from an acute to a sub-acute phase in 24 h, and *ASIC3*^-/- ^mice could have recovered faster from hyperalgesia. Although ASIC3 is believed to play a more important role in secondary hyperalgesia (hypersensitivity occurred in non-injured sites) induced by inflamed muscle or joints than primary hyperalgesia in inflamed tissues [16,17], in this study we provide evidence that ASIC3 does play a role in subcutaneous primary hyperalgesia in later stages. This conclusion is also supported by a recent report showing that subcutaneous injection of the ASIC3-selective blocker APETx2 can alleviate the inflammation-induced hyperalgesia [22]. However, the role of ASIC3 for sub-acute-phase primary hyperalgesia is not applied to the deep tissues, because a recent study reported that *ASIC3*^-/- ^mice did not show different levels of primary hyperalgesia in inflamed joint induced by 3% of carrageenan than *ASIC3*^+/+ ^mice [17].

We did not observe a significant difference in effects of inflammation between *ASIC3*^+/+ ^and *ASIC3*^-/- ^mice on pathological examination, which indicates that ASIC3 does not play a role in the development of inflammation, and the difference in hyperalgesia may not be related to the degree of inflammation.

During inflammation, the enhanced expression of ion channels sensitizes primary afferent neurons and produces hyperexcitability, thereby producing hyperalgesia. Up-regulation of ASIC3 and sodium channels during inflammation have been documented [8-10,23-25]. However, real-time PCR results in our study showed no transcriptional change in *ASIC3*, *Nav1.6*, *Nav1.7*, and *Nav1.8 *mRNA level following inflammation. These results are contradictory to those in previous studies. The only up-regulated gene we found was *Nav1.9*, an ion channel reported to be unchanged under intraplantar carrageenan-induced inflammation [25]. *Nav1.9 *mRNA level has been shown to increase by day 7 with CFA-induced inflammation [26]; however, its expression was not changed in our CFA model. A possible explanation for the discrepancy between our study and previous studies is the difference in sampling and signal quantification. Previous studies used methods such as RT-PCR, *in situ *hybridization or immunostaining, which were semi-quantitative. Furthermore, both *in situ *hybridization and immunostaining examine one plane of cells, whereas with real-time PCR, the total mRNAs from all cellular populations in a single DRG were quantified. Lack of transcriptional change in a single DRG by real-time PCR cannot rule out the possibility of up- or down-regulated genes in subgroups of cells or involvement of the gene in posttranscriptional regulation for the process of sensitization. Another issue to consider is the timing of sampling, since regulation of these genes might be transient and time dependent. Animal species and genetic background may also account for the discrepancies to a certain extent, because mice express relatively less ASIC channels in DRGs than do rats [27,28].

The up-regulation of *Nav1.9 *on day 2 with intraplantar carrageenan-induced inflammation was significant and ASIC3 dependent. The functional importance of *Nav1.9 *in modulation of pain behavior in inflammation has been previously investigated by disrupting the ion channel in mice [29]. Mechanical and thermal thresholds are comparable between *Nav1.9*^-/- ^and wild-type mice in the absence of injury. In contrast, inflammation-mediated pain behavior differs prominently in *Nav1.9*^-/- ^mice as compared with wild-type mice. Intraplantar injection of carrageenan induced thermal hyperalgesia in both wild-type and *Nav1.9*^-/- ^mice in the first 3 h post-injection; however, the hyperalgesia was diminished 24 h later in *Nav1.9*^-/- ^mice. This finding matches our observation of *ASIC3*^-/- ^mice with longer paw withdrawal latency starting at 4 h and continuing through day 2, which is indicative of attenuated thermal hyperalgesia.

The similar phenotypes in the pain behavior for *Nav1.9*^-/- ^and *ASIC3*^-/- ^mice under inflammation and the ASIC3-dependent up-regulation of *Nav1.9 *suggest that inflammation induces tissue acidosis, which leads to activation of ASIC3 and subsequent ASIC3-dependent up-regulation of *Nav1.9*; the increased *Nav1.9 *level in turn contributes to thermal hyperalgesia. In the absence of ASIC3, *Nav1.9 *cannot be up-regulated, and thermal hyperalgesia becomes attenuated.

In contrast to up-regulation of *Nav1.9 *transcripts, increase of I_Nav1.8 _on day 2 with intraplantar carrageenan-induced inflammation was significant and ASIC3 dependent on electrophysiology (Fig. 11). Although our electrophysiolgical data did not show increased I_Nav1.9 _in overall small to medium neurons to support the up-regulation of *Nav1.9 *transcripts during inflammation, we did find a significant increase of I_Nav1.9 _in neurons that did not express Nav1.8 activity in *ASIC3*^+/+ ^mice (65.4 ± 16.0 vs. 506.5 ± 147.7 pA for the control vs. carrageenan group, respectively, P < 0.05). Further study would aim to determine whether a specific subset of DRG neurons plays an important role in regulating the ASIC3-dependent effect during inflammation. However, the increase in I_Nav1.8 _was robust, with an increase of up to threefold in peak amplitudes, which was associated with maximal mechanical and thermal hyperalgesia on day 2 of carrageenan inflammation. However, a previous study reported slight change of I_Nav1.8 _peak amplitude on day 4 of carrageenan inflammation, when the *Nav1.8 *mRNA was significantly up-regulated and the current density of I_Nav1.8 _was slightly increased [24]. Thus, the alteration of Nav1.8 activity during inflammation may be time-dependent, as was seen in many studies [25,30]. Nevertheless, the increase of I_Nav1.8 _is intriguing because Nav1.8 is known to play a role in mechanical nociception, and inhibition of Nav1.8 would abolish inflammation-induced mechanical and thermal hyperalgesia [31,32]. Therefore, the increase in Nav1.8 activity may account in part for the carrageenan-induced mechanical hyperalgesia found in *ASIC3*^+/+ ^mice but not in *ASIC3*^-/- ^mice.

### Intramuscular inflammation

*ASIC3 *is critical for the development of secondary mechanical hyperalgesia with muscle inflammation [16]. Our findings agree with this result and show *ASIC3*^+/+ ^mice with significant mechanical hyperalgesia 2 days after intramuscular carrageenan injection. However, we did not observe thermal hyperalgesia in either genotype with carrageenan-induced inflammation as was reported for C57BL6 mice [16]. Perhaps the time to establish secondary thermal hyperalgesia in CD1 mice is longer than is needed in C57BL6 mice.

In inflamed muscle, *ASIC3*^-/- ^mice seemed to display milder pathological features, including infiltration of leukocytes, formation of granulomas and vasculitis, than *ASIC3*^+/+ ^mice. Previous reports described the same pathological responses in both genotypes, and an assessment of neutrophilic activity by myeloperoxidase in carrageenan-inflamed muscle also suggested that ASIC3 played no role in the development of inflammation [16]. Although neutrophilic activity represents only one aspect of inflammation, the type of immune cells recruited and their organization and behavior vary by inflammatory conditions. As we examined closely, the inflammation processes involved in CFA- or carrageenan-induced inflammation are not simple. Carrageenan-induced vasculitis was not documented, although in one study histological examination was carried out to characterize the transition of immune cell-type from acute to chronic inflammation of the rat leg muscle [33]. Also, CFA has not been used as a muscle inflammation model; thus, no report of its effect on granuloma formation in muscle exists.

The development of granulomas and vasculitis involves complicated interaction among immune cells, cytokines and chemokines. Granulomas are the aggregation of mononuclear inflammatory cells and modified macrophages organized in a compact nodule, with involvement by a complicated interaction between antigen-presenting macrophages and T lymphocytes mediated by various cytokines [34]. However, the pathogenesis of vasculitis is still not well understood, but endothelium injury is generally believed to be the fundamental event in its development. Responding to neuropeptides (substance P or calcitonin gene-related peptide), endothelial cells mediate several features of chronic inflammation such as vasodilatation, leukocyte migration, cytokine production and cellular adhesion molecule expression [35]. During vascular tone regulation and interaction with immune cells, damage to endothelial cells could occur [36].

The involvement of ASIC3, an ion channel present on neurons, in the pathogenesis of these features is thus intriguing. The interplay between the neuronal and immune system has become an interesting topic recently; body systems do not work alone. Neurons could initiate a cascade of cytokine synthesis and release and recruitment of inflammatory machinery. The process is neurogenic inflammation, whereby small-diameter sensory neurons release neuropeptides such as substance P and calcitonin-gene related peptide upon activation. Blocking nerves results in decreasing the inflammation consequences of carrageenan-induced inflammation [37]. ASIC3 could thus participate in the pathogenesis of granuloma formation and vasculitis through activating neurogenic inflammation. Lacking ASIC3 may alter the properties of sensory neurons [38], thus influencing its neurogenic release and the inflammatory consequences. This point should be further investigated. However, the immune system could also affect how the neural system perceives pain through activated mast cells, macrophages, neutrophils, and the cytokines they release [1]. ASIC3-mediated inflammation could be essential for the hyperalgesia we observed in wild-type mice.

## Conclusion

Here we systematically characterized changes in behavior, pathology, gene expression, and TTX-resistant sodium currents following inflammation induced by CFA and carrageenan in *ASIC3*^-/- ^and *ASIC3*^+/+ ^mice and showed that the deletion of the *ASIC3 *gene affected features in both inflammation models. The *ASIC3*^-/- ^mice displayed milder symptoms of inflammation than the *ASIC3*^+/+ ^mice. These findings suggest that ASIC3 mediates the sub-acute phase of primary hyperalgesia produced by inflammation, and the ASIC3-mediated responses to inflammation in the paw differ from those in muscle. ASIC3 participates in the development of sub-acute-phase hyperalgesia by up-regulating *Nav1.9 *and TTX-R currents in subcutaneous inflammation and mediates the process of granuloma formation or vasculitis in muscular inflammation.

## Methods

### Animals

Adult (8- to 12-week-old) female CD1 mice were kept in a 12 h light-dark cycle with sufficient water and food. *ASIC3*^-/- ^mice were produced as described [15] and backcrossed with CD1 mice for more than 10 generations [28]. *ASIC3*^+/+ ^and *ASIC3*^-/- ^mice were offspring of heterozygous (*ASIC3*^+/-^) intercrosses. The behavioral experiments and examination of pathology were conducted blinded to animal genotype. All experimental protocols were approved by the Institute of Animal Care and Use Committee of Academia Sinica. Animals were bred and taken care of in accordance with the current Guide for the Use of Laboratory Animals (National Academy Press, Washington DC).

### Inflammatory models

*ASIC3*^+/+ ^(n = 72) and *ASIC3*^-/- ^(n = 73) mice were divided into 6 groups. Under anesthesia with 2% halothane (Halocarbon laboratory), 3 groups (~18 mice for each) of each genotype were given a single injection of 20 μl saline (pH 7.4, buffered with 20 mM HEPES to rule out the effects of acidic saline), CFA (0.5 mg/ml heat-killed *M. tuberculosis *[Sigma, St. Louis, MO] suspended in oil:saline 1:1 emulsion) or 3% carrageenan (lambda carrageenan, type IV; Sigma) in the plantar surface of the left hind paw (Day 0) to induce intraplantar inflammation. Behavioral assessments were conducted at 4 h, 1 day, and 2 and 7 days after induction of inflammation, and DRG were harvested at 4 h and days 2 and 7. For this purpose, 6 mice from each group were tested for mechanical responses after 4 h, and then killed; another 6 mice, were tested for behavioral response to pain at days 0, 1 and 2, and then killed at day 2; another 6 mice, for thermal responses 4 h after injection and again for both behavioral assessments at day 7. The other 3 groups (6 mice for each group) of each genotype received an injection of saline, CFA or carrageenan in the left gastrocnemius muscle belly to induce intramuscular inflammation. Behavior was tested at days 0, 1 and 2, and mice were killed on day 2.

### Assessment of thermal and mechanical hyperalgesia

Thermal and mechanical sensitivities were tested prior to (day 0) and at 4 h and days 1, 2, and 7 following injections. All tests were performed at constant room temperature of 24°C, and stimuli were applied only when mice were calm but not sleeping or grooming. If both tests were performed on the same day, mechanical responses were first assessed, and then thermal tests were performed at least 1 h later.

For thermal sensitivity assessment, mice were moved to a small cubicle on a glass platform and allowed to habituate for 1 h. The radiant heat source was directed to the plantar surface of one hind paw through the glass until the mouse withdrew its paw. The latency of paw withdrawal from the onset of stimulation was measured sequentially for both hind paws by use of an IITC analgesiometer (IITC Life Sciences, Woodland Hills, CA). The light intensity was set to obtain a baseline response time of approximately 10 sec. The cut-off time was set to 30 sec to minimize heat damage to the skin. Sensitivity of each hind paw was measured with 3 trials, with a 10-min recovery period between each trial. The response was determined by the paw withdrawal latency defined by the mean from 3 trials at each time point.

Mechanical sensitivity was measured by testing the number of responses to stimulation with five applications of von Frey filaments. Mice were placed on an elevated wire mesh platform in a plexi-glass chamber and allowed 1 hr for acclimatization. A von Frey filament of 0.02 g bending force was used as a baseline stimulation. Filaments were briefly applied 5 times to each hind paw, with a 30-sec interval between each application. A response was considered valid with an abrupt foot lift on application of the von Frey filament.

### Examination of pathologic specimens

Mice were sacrificed by use of CO_2_, and paw thickness was measured at the metatarsal level. The inflamed left hind paw or leg muscle was dissected, fixed in 4% paraformaldehyde overnight and then embedded in paraffin. Paws were decalcified before embedding. Sections were deparaffinized and underwent H&E staining. All sections were examined under a light microscope independently by two pathologists. The inflamed muscle sections were further analyzed by determining the number of granulomas or the percentage of vessels showing vasculitis. For CFA-inflamed muscle, the total area of the muscle cross-sections was determined by use of ImageProPlus software, and the number of well-formed granulomas was counted. A well-formed granuloma was defined as a compact, circumscribed structure with epithelioid macrophages. Vascular changes, including integrity of endothelial lining and vessel wall, perivascular extravasation of erythrocytes, in carrageenan-inflamed muscle were inspected. Infiltration of inflammatory cells, swelling of endothelial cells and apoptotic bodies were used to define vasculitis. These studies were conducted blinded to the genotype of the mice.

### Immunocytochemistry

Mice were anesthetized with an overdose of pentobarbital and intracardially perfused with normal saline followed by 4% paraformaldehyde. The left gastrocnemius muscle was immediately dissected and post-fixed with 4% paraformaldehyde. Post-fixed tissues were placed in 30% sucrose (in pH 7.4 PBS) overnight, then embedded in OCT and rapidly frozen with use of liquid nitrogen and stored at -80°C. Frozen sections were cut 20-μm thick on a cryostat and mounted on glass slides. Slides were incubated with blocking solution containing 3% BSA, 0.1% Triton X-100, and 0.02% sodium azide in PBS for 90 min at room temperature. After blocking, slides were incubated with primary antibodies (rabbit anti-ASIC3, 1:500, Alomon Lab, Jerusalem, Israel; guinea pig-anti-PGP9.5, 1:500, Chemicon, Temecula, CA) prepared in blocking solution at 4°C overnight. Slides were visualized by use of fluorescence-conjugated secondary antibodies (goat anti-rabbit-Alexa Fluor-488 or goat anti-guinea pig-Alexa Fluor 594, 1:200, Invitrogen, Carlsbad, CA) and mounted on cover slips with DAPI-containing mounting medium (Vector, Burlingame, CA). The ASIC3 staining was eliminated when the control peptides (provided by the manufacture) were applied (data not shown).

### Gene expression in DRG

Left and right L1-L5 DRGs were harvested separately and stored at -80°C. L1 DRGs were defined as those posterior to the last pair of ribs. Total RNAs of DRGs were isolated by use of the RNeasy mini kit (Qiagen, Valencia, CA) according to the manufacturer's instructions. Each RNA sample was eluted in 30 μl DEPC-treated water. 11 μl from each RNA sample was mixed with 1 μl oligo(dT)_18 _(250 ng) and 1 μl 10 mM dNTP mix (GeneMark, Taipei, Taiwan), heated to 65°C for 5 min and incubated on ice for at least 1 min. 4 μl 5× First-Strand Buffer, 1 μl of 0.1 M DTT, 1 μl RNaseOUT and 1 μl SuperScript III Reverse Transcriptase (Invitrogen, Carlsbad, CA) were then added to the mixture and incubated at 50°C for 1 h for reverse transcription (RT). The reaction was inactivated at 70°C for 15 min. For real-time PCR, 12.5 μl of 2× SYBR Green PCR Master Mix (ABI, Foster City, CA), and 0.5 μl of the desired primer mixture were added to the RT cDNA templates to a final volume of 25 μl. PCR involved the ABI prism 7700 sequence detector for 95°C for 10 min, followed by 40 cycles of 95°C for 15 sec, and 60°C for 1 min. The primer sequences were as follows:

ASIC3: F 5'- CCCAGTCCGACTTTTGACAT - 3'

R 5'- CAGAGTTGAAGGTGTAGCAT - 3'

Na_v_1.6: F 5' - CCGATGGAAGAACGTCAAGA - 3'

R 5' - ATAATCAGGCTGCTCGTCCG - 3'

Na_v_1.7: F 5'- GACCTTGGCCCCATTAAATCT - 3'

R 5'- CTTGCCAGCAAACAGATTGAC - 3'

Na_v_1.8: F 5' - GTAGTGGTGGATGCCTTGGT - 3'

R 5'- AAGTGGCCGGTATTGTTTTG - 3'

Na_v_1.9: F 5'- GATGTGCCCAAGATCAAGGT - 3'

R 5'- TTCCGACGTTCAATCTTTCC - 3'

TRPV1: F 5' - TCTCCACTGGTGTTGAGACG

R 5' - GGGTCTTTGAACTCGCTGTC

GAPDH: F 5'- GGAGCCAAACGGGTCATCATCTC - 3'

R 5' - GAGGGGCCATCCACAGTCTTCT - 3'

### Electrophysiology

DRG neurons of lumbar 5 segments were isolated from mice treated with intraplantar saline or carrageenan for 2 days. DRG acute culture and settings for whole-cell patch recording were as previously described [28]. The internal solution contained (in mM) 10 NaCl, 110 CsCl, 20 tetraethylammonium-Cl, 2.5 MgCl_2_, 5 EGTA, 3 Mg^2+^-ATP, and 5 HEPES, adjusted to pH 7.0 with CsOH. The external solution contained (in mM) 100 NaCl, 5 CsCl, 30 tetraethylammonium-Cl, 1.8 CaCl_2_, 1 MgCl_2_, 0.1 CdCl_2_, 25 glucose, 5 4-aminopyridine, and 5 HEPES, adjusted to pH 7.4 with HCl. Osmolarity was adjusted to 290 mosmol/kg with glucose. Before whole-cell patch recording, cells were stained for isolectin B4(IB4)-FITC (4 μg/ml) for 2 min, and only small- to medium-size (< 34 μm) DRG neurons were selected for recording [28]. Recordings were performed in external solution containing 200 nM TTX (Tocris, Avonmouth, UK). TTX-resistant sodium currents were evoked by a 30-ms test pulse between -70 and 0 mV in 10-mV steps from a holding potential of -80 mV [20]. For clarity, only the -60 and -40 mV current traces were shown. All recording was at room temperature (21–25°C) and completed within 24 h after plating. A total of 144 DRG neurons were patch recorded; half of them were IB4-positive neurons.

### Statistics

Data are expressed as mean ± SEM. Analysis involved use of SAS9.1 (SAS Inst, Cary, NC). Radiant heat testing was analyzed by two-way ANOVA followed by least squares means post-hoc test. Mann-Whitney U-test was used to determine differences in mechanical sensitivity between groups and time points, differences in paw thickness, granuloma, vasculitis, and quantitative PCR. Mean peak amplitudes of TTX-R currents were compared between groups via one-way ANOVA. The level of significance was set at P < 0.05.

## Abbreviations

ASIC3: acid-sensing ion channel 3; DRG: dorsal root ganglion; CFA: complete Freund's adjuvant; Nav: voltage-gated sodium channel; TRPV1: capsaicin receptor; TTX: tetrodotoxin.

## Competing interests

The authors declare that they have no competing interests.

## Authors' contributions

YTY performed pain behavior testing, pathological examination, and quantitative PCR. PHT and STH examined and interpreted the pathological data. CJC performed mouse genotyping, analyzed the pathological & behavioral data, and performed immunocytochemistry. YWL performed the electrophysiology. CCC oversaw the research and prepared the manuscript with help from all others.

## Supplementary Material

Additional file 1 **Inflammation-mediated hyperalgesia in the contralateral paws.** A, Thermal hyperalgesia results of intraplantar inflammation. B, von Frey filament test results of intraplantar inflammation. C, Thermal hyperalgesia results of intramuscular inflammation. D, von Frey filament tests results of intramuscular inflammation. WT: *ASIC3*^+/+ ^mice, n = 6 in each group. KO: *ASIC3*^-/- ^mice, n = 6 in each group. *p < 0.05 compared to baseline latency. #p < 0.05 comparison between *ASIC3*^+/+ ^and *ASIC3*^-/- ^groups. Click here for file

Additional file 2 **Gene expression level in *ASIC3*^+/+ ^DRG 2 days after induction of inflammation.** A, Expression of *ASIC3*, *Nav1.6*, *Nav1.7*, *Nav1.8*, *Nav1.9 *and *TRPV1 *2 days after intraplantar carrageenan-induced inflammation in L5 DRG. B, Expression of *ASIC3*, *Nav1.6*, *Nav1.7*, *Nav1.8*, *Nav1.9 *and *TRPV1 *2 days after intramuscular inflammation induction in L4 DRG. *p < 0.05 compared with saline group of the same side. Click here for file
