# Supplementary figures and images for: Role of acid-sensing ion channel 3 in sub-acute-phase inflammation
Source: Mol Pain. 2009 Jan 7;5:1. doi: 10.1186/1744-8069-5-1 (PMC2632618; doi:10.1186/1744-8069-5-1)

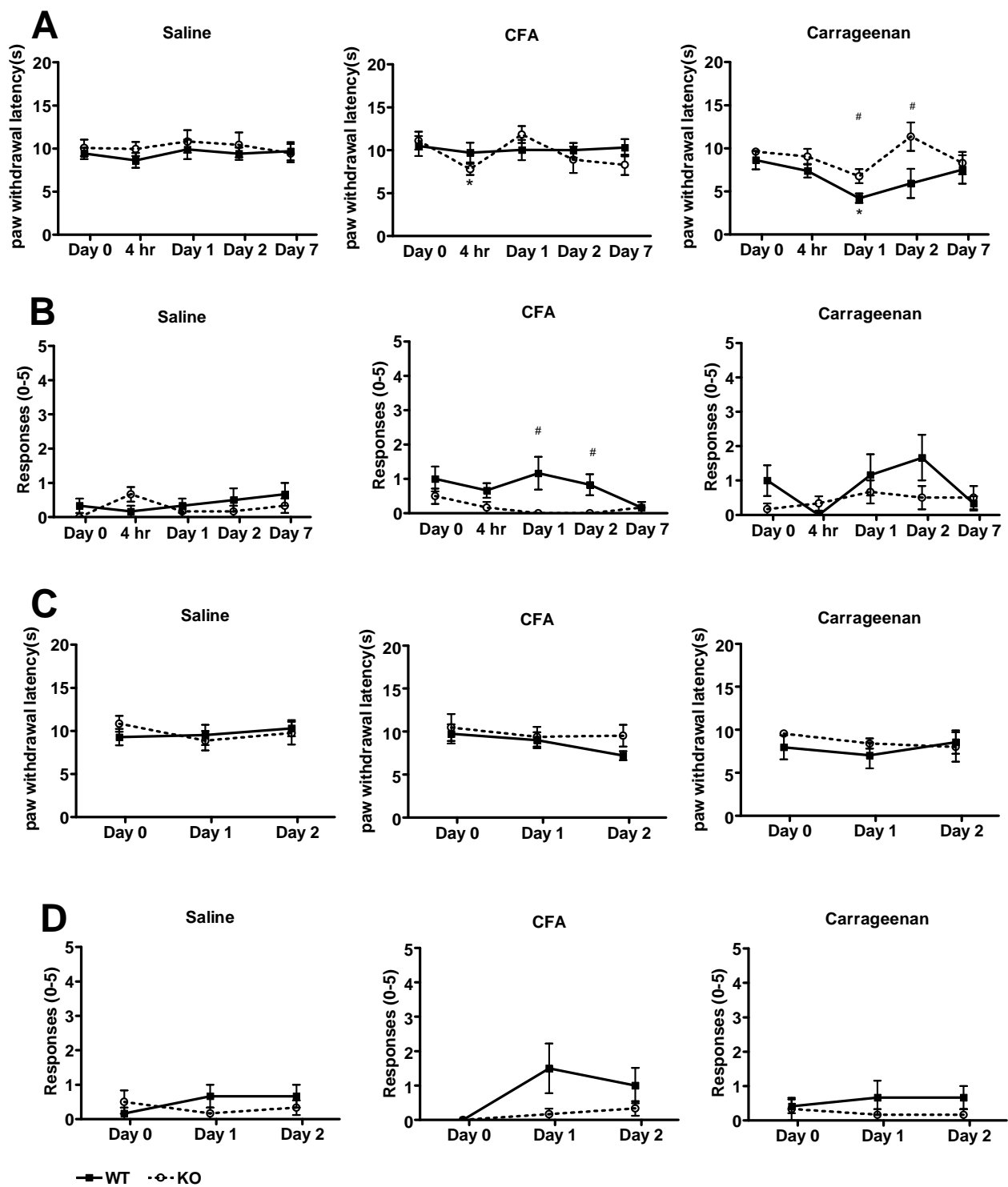

Supplement: Additional file 1 — Inflammation-mediated hyperalgesia in the contralateral paws. A, Thermal hyperalgesia results of intraplantar inflammation. B, von Frey filament test results of intraplantar inflammation. C, Thermal hyperalgesia results of intramuscular inflammation. D, von Frey filament tests results of intramuscular inflammation. WT: ASIC3+/+ mice, n = 6 in each group. KO: ASIC3-/- mice, n = 6 in each group. *p < 0.05 compared to baseline latency. #p < 0.05 comparison between ASIC3+/+ and ASIC3-/- groups. [file 1744-8069-5-1-S1.pdf]

**A**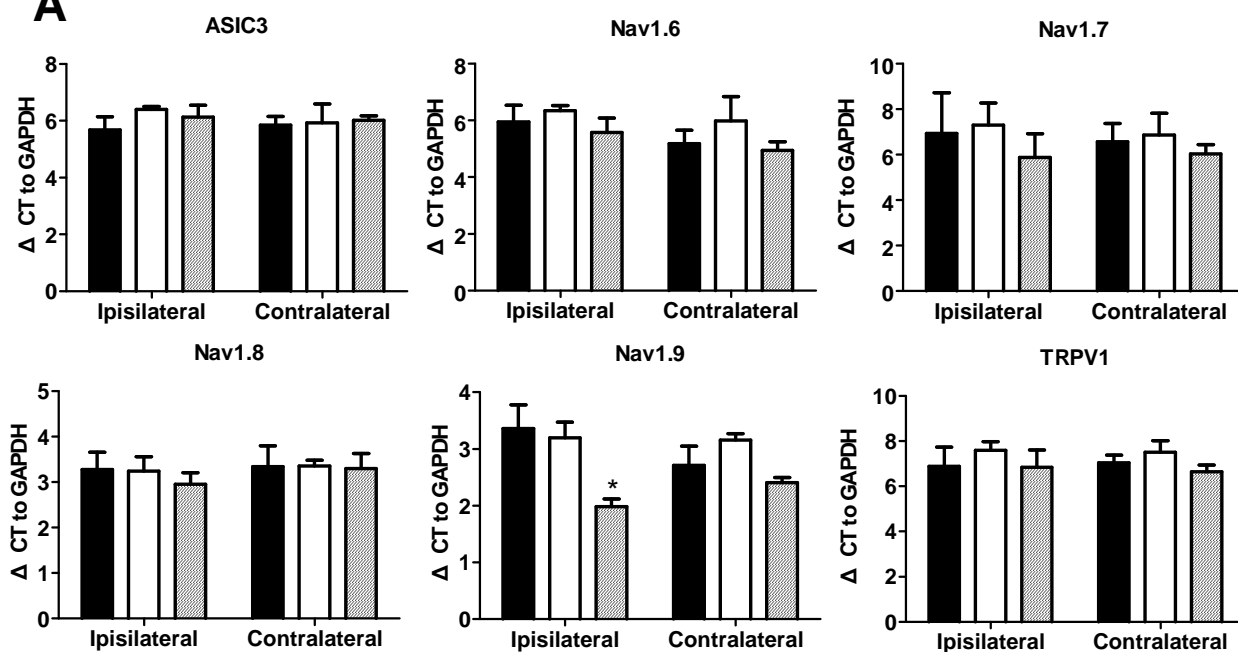**B**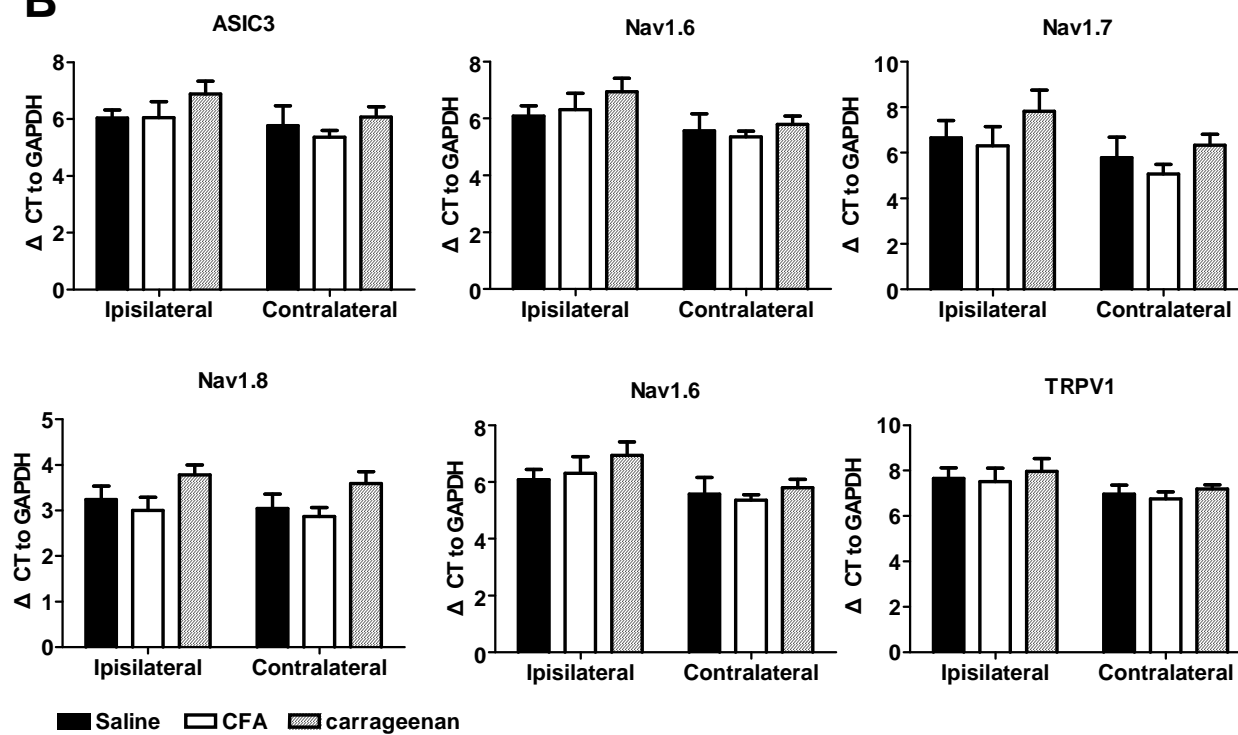

Supplement: Additional file 2 — Gene expression level in ASIC3+/+ DRG 2 days after induction of inflammation. A, Expression of ASIC3, Nav1.6, Nav1.7, Nav1.8, Nav1.9 and TRPV1 2 days after intraplantar carrageenan-induced inflammation in L5 DRG. B, Expression of ASIC3, Nav1.6, Nav1.7, Nav1.8, Nav1.9 and TRPV1 2 days after intramuscular inflammation induction in L4 DRG. *p < 0.05 compared with saline group of the same side. [file 1744-8069-5-1-S2.pdf]
